# The Impact of a Simplified Hydrostatic Bypass Flow Technique on Error Detection during Surgical Limb Revascularization

**DOI:** 10.3390/jcm9041079

**Published:** 2020-04-10

**Authors:** Anita Rybicka, Paweł Rynio, Rabih Samad, Halina Szumiłowicz, Paweł Szumiłowicz, Sebastian Kazimierczak, Tomasz Zakrzewski, Piotr Gutowski, Elżbieta Grochans, Agata Krajewska, Arkadiusz Kazimierczak

**Affiliations:** 1 Department of Nursing, Faculty of Health Sciences, Pomeranian Medical University, Szczecin, Żołnierska 48, 71-210 Szczecin, Poland; grochans@pum.edu.pl; 2 Vascular Surgery Department, Pomeranian Medical University, Powstańców, Wielkopolskich 72, 70-111 Szczecin, Poland; ryniopawel@gmail.com (P.R.); rasamad@wp.pl (R.S.); isz@life.pl (H.S.); szumilp@o2.pl (P.S.); zakrzewski.t@wp.pl (T.Z.); piotr_gutowski@poczta.onet.pl (P.G.); biker2000@icloud.com (A.K.); 3 Anaesthesiology, Perioperative Care and Pain Therapy Department, Helios Hospital in Berlin-Buch, Schwanenbecker Chaussee 50, 13125 Berlin, Germany; sebastian.kazimierczak@helios-kliniken.de; 4 Department of Neurology, Pomeranian Medical University, Unii Lubelskiej 1, 71-210 Szczecin, Poland; agattw@gmail.com

**Keywords:** chronic limb-threatening ischemia, auto-vein bypass, error detection, blood flow measurement

## Abstract

Technical errors have an impact on the results of surgical lower limb revascularization. Use of ultrasound scanning or angiography on the operating table is inconvenient and, in case of angiography, carries a certain risk of radiation and contrast exposure. A simpler method of screening for errors is required. This study assessed the accuracy of a new simple hydrostatic bypass flow technique during surgical limb revascularization. In all, 885 patients were included in the retrospective study. All were treated for Chronic Limb-Threatening Ischemia (CLTI) with a femoropopliteal bypass. Preoperatively, the radiological Vascular Surgery/International Society of Cardiovascular Surgery (SVS/ISCVS) score was used to assess the complexity of the anatomical changes. The surgeon made a subjective runoff assessment for every surgery. In 267 cases, the hydrostatic bypass flow (HBF) technique was used, and, in 66 cases, a digital subtraction angiography (DSA) was used. In each case, a postoperative Doppler ultrasound (DUS) examination was performed following the HBF. Good early results were achieved in 89.46%, and 154 errors (17.4%) were detected (85 were detected on the operating table, including 57 technical errors). Independent efficacy in error detection was proven with a postoperative Doppler examination (Aera Under Curve (AUC) = 0.89; criterion mid-graft peak systolic velocity (PSV) <24 cm/s, *p* = 0.00001) and hydrostatic bypass flow (AUC = 0.71, criterion HBF < 53 mL/min, *p* = 0.00001) during surgery. The hydrostatic bypass flow technique is an effective intraoperative screening method in bypass surgery. Algorithmic use of HBF, DSA if needed, and DUS postoperatively improves the outcome. HBF sufficiently reduced the need for on-table angiography.

## 1. Introduction

Bypass surgery remains a gold standard for lower limb revascularization in the case of complex anatomical changes (Global Limb Anatomic Staging System, GLASS III) based on Global Vascular Guidelines (GVG), provided the operative risk is acceptable or if endovascular treatment has failed [1,2]. Technical errors are the most important factor impacting the results of bypass surgery [3,4]. Therefore, the intraoperative use of angiography, angioscopy, and sonography can improve the results [4,5]. The runoff can be predicted using the SVS/ISCVS score [2,6]. Attempts to correlate the directly measured bypass flow and the radiologically assessed runoff in the SVS/ISCVS score have been unsuccessful [7]. Currently the most specific method of error detection is intraoperative angiography [3,4,5,8,9]. Unfortunately, angiography does not accurately assess the resistance and flow in the bypass. This is why, many authors suggest using an intraoperative ultrasound [4,10,11]. However, intraoperative angiography and sonography are time consuming and potentially risky. In turn, subjective methods (e.g., back-flow assessment or flushing the bypass using a syringe, pulse palpation etc.) are the easiest, but are based only on the “gut feeling” of the surgeon and are therefore not recommended. In light of the above, we have introduced a new simple and objective method called HBF (hydrostatic bypass flow), followed by an algorithmic use of on-table angiography and a Doppler examination performed after the operation. This paper presents the final results of our study.

## 2. Aim

The purpose of this study was to examine the accuracy of a new simple hydrostatic bypass flow technique during surgical limb revascularization.

## 3. Methods

This was a single-center, nonrandomized, retrospective study. Inclusion criteria: All patients who underwent a below-the-knee bypass surgery with an autologous vein due to critical limb ischemia (as of 2019—chronic limb-threating ischemia) [2]. These were patients in stage 4, 5, or 6 of the Rutherford Classification. Patients with a need for prosthetic bypass were excluded from the study. All of the patients were asked to provide written informed consent allowing their clinical data to be used for scientific research. The procedures were followed in accordance with the ethical standards of the local bioethical committee and the Helsinki Declaration (Bioethics’ Committee approval: 14/KB/V/2008, OIL, Szczecin). Revascularization was performed according to TransAtlantic Inter-Society Consensus (TASC) guidelines (mostly TASC D after a failure of an endovascular attempt, which corresponds to stage 3 of the current GLASS (Global Limb Anatomic Staging System) and a risk of death during surgery below 5%) [1,2,12,13]. Anatomic eligibility was based on a CT angiogram. A venous below-the-knee bypass was performed to restore an adequate target arterial pathway [2]. Epidural or subdural analgesia was preferred during surgery. Heparin 0.5–1 mg/kg iv was given during surgery followed by Low-Molecular-Weight Heparin (LMWH) 0.5 mg/kg until discharge. On discharge we prescribed a daily dose of 75 mg of acetylsalicylic acid to every patient. Before the operation, the expected runoff was calculated using the SVS/ISCVS radiological score in each case. During the operation, we used three methods to assess the actual runoff vs. subjective runoff (based on the surgeon’s “gut feeling”) in each case. In the examined group, we used hydrostatic bypass flow and on-table angiography (if required), followed by a postoperative ultrasound scan. We looked for any errors that could potentially lead to a revascularization failure. There was no randomization and no head-to-head comparison of the above methods. Data collection for the control group started in 2011 and ended in 2016 (only subjective runoff assessment). Data for the examined group were collected between 2016 and 2018.

### 3.1. Subjective Runoff Assessment (SRA)

**Figure 1:**
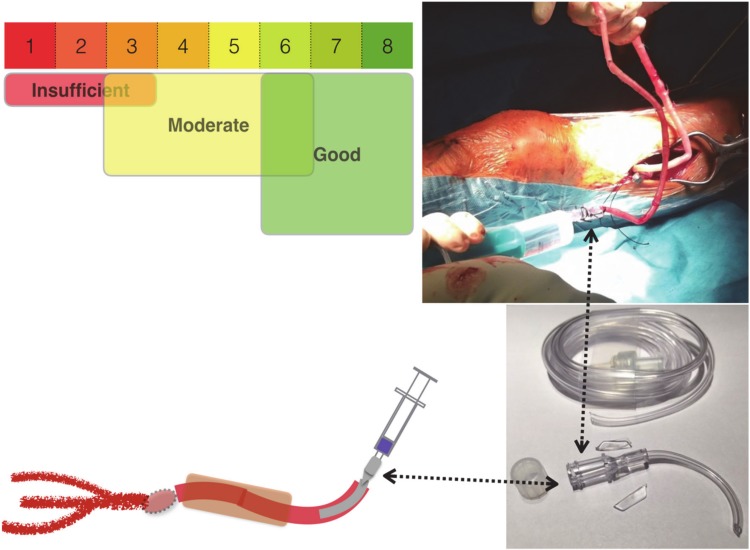
Subjective runoff assessment (SRA).

SRA was based on the surgeon’s “gut feeling”. After performing a distal anastomosis, the surgeon assessed the flow and resistance by flushing the bypass with 20 mL of 0.9% NaCl. The measurement of SRA was reported on a numeric scale (1–8). In order to “feel” the resistance, a low-resistance catheter for vein cannulation was used (see Figure 1). This cannula allows at least 1000 mL saline per minute to pass through (Margomed, Lublin, Poland; certificate-CE0434; external diameter 2.1 mm; internal diameter 0.55 mm). We used a cannula, 3 cm in length (Figure 1).

The figure shows the equipment that is necessary for the cannulation of the vein when harvesting it for the bypass surgery. The cannula is easy to prepare by cutting off a 3 cm segment of a standard cannula. The set can be used by the surgeon for flushing the bypass and checking the outflow (subjective scale: good, moderate, or poor) and, if needed, for hydrostatic bypass flow assessment.

### 3.2. Hydrostatic Bypass Flow

**Figure 2:**
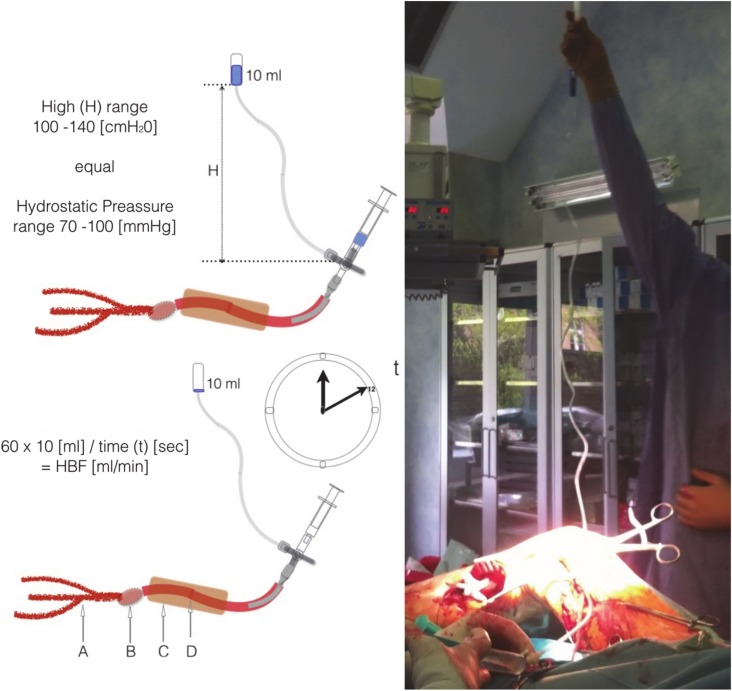
Hydrostatic bypass flow.

Hydrostatic bypass flow (HBF) uses the same cannula as SRA but expands it with an extra tap and drip (see Figure 2). The measurement of the HBF is based on a simple calculation: HBF = 60/t × 10 [mL/min]. Where (t) is the transfusion time of 10 mL of normal saline through the bypass under the hydrostatic pressure. The hydrostatic pressure is around 70–100 mmHg and is produced by a water column (H) in the drip. A water column of 100 cm produces about 70 mmHg of pressure (1 mmHg = 13.6 cm H_2_O). To perform the measurement, the container of the drip must be prefilled with 10 mL saline.

The figure shows how to perform the HBF measurement. The vein cannula must be connected to a standard drip. Raising the drip container at least 1 m creates appropriate hydrostatic pressure. Starting the stopwatch while flushing the bypass helps to assess the crucial parts of vascular reconstruction. It makes it possible to:

(A) assess the peripheral arteries (resistance of target vessels—peripheral runoff, their stenosis or occlusion, lack of target vessels, dissection, embolization, thrombosis),
(B) assess the quality of the anastomosis (technical error, stenosis, dissection),
(C) monitor the bypass channel (possible compression, entrapment),
(D) verify the bypass patency itself (dissection, torsion, kinking, stenosis, thrombosis).

### 3.3. Doppler Screening (DUS)

Ultrasound screening was performed after the surgery. Mid-graft peak systolic velocity (PSV) as well as lesion PSV and PSV ratio were measured. According to the GVG guidelines, the criteria suggestive of impaired flow were mid-graft PSV < 45 cm/s, lesion PSV >300 cm/s, and PSV ratio >3.5 [2]. We also measured the diameter of the bypass (mm) along the whole graft. Siemens Medical Model KT-LM200HDS was used. A detailed Doppler examination was performed only in cases of flow impairment.

### 3.4. Digital Subtraction Angiography

**Figure 3:**
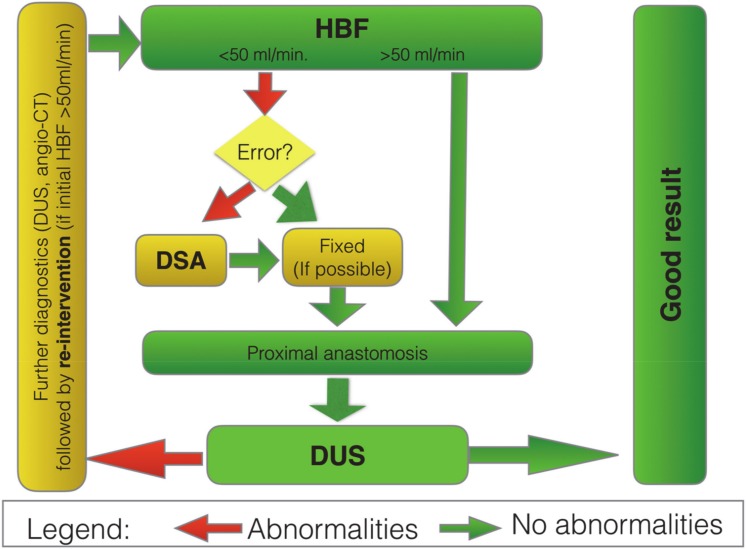
Bypass screening algorithm in the examined group. HBF—hydrostatic bypass flow; DSA—digital subtraction angiography; DUS—Doppler ultrasound.

Digital Subtraction Angiography (DSA) was used if the HBF measurement revealed any flow impairment and the surgeon did not recognize the problem right away. As presented in the algorithm in Figure 3. We used a mobile-C-Arm (Ziehm Vision RFD Imaging GmbH. Donaustrasse 31, 90451 Nuremberg, Germany; serial number 20498). The mean volume of the contrast was 15 mL (Visipaque; Joxidanolum 652 mg/mL, GE-Healthcare AS, Oslo, Norway). Subjective runoff assessment (SRA) was used in the control group. In the examined group, DSA, HBF, and DUS were used algorithmically (Figure 3).

### 3.5. HBF—Hydrostatic Bypass Flow, DSA—Digital Subtraction Angiography

The figure presents a simple algorithm for the necessary interventions, depending on the results of the HBF. If the HBF is satisfactory (>50 mL/min), there is no need for on-table angiography. If, after surgery, thrombosis of the bypass is detected, then re-intervention has a high chance of success. 

Initial results were considered good if the ultrasound scanning confirmed the patency of the bypass and the patient survived without an extensive amputation. If the bypass was patent, but the patient still lost a limb, the result was considered bad. Any additional worsening of the clinical status or a definite lack of clinical improvement would also be recognized as a bad result. We did not apply the standard rule to alleviate the patients’ condition by reducing their condition by at least one stage in the Rutherford classification, because in advanced cases (Rutherford 6) a change in the clinical status would require more time. An eligibility error was detected if revascularization was unsuccessful and no technical errors were detected, or if there was a severe cardiovascular complication not related to a technical failure.

## 4. Statistics

Analysis was performed using Statistica.pl, version 10.0 (Stat Soft, Inc., Palo Alto, CA, USA). Multifactorial discriminant analysis was performed using the Wilks’ lambda test. The predictive value was assessed using receiver operating curve (ROC) analysis. The area under the curve (AUC) was calculated, and criteria together with sensitivity and specificity were assessed. Differences were considered significant if the *p*-value was less than 0.05.

## 5. Material

The study was carried out from 2008 to 2015 at the Vascular Surgery Department of the Pomeranian Medical University in Szczecin, Poland. A group of 885 patients underwent a femoropopliteal bypass surgery to treat chronic limb-threatening ischemia. Epidemiology of the group is presented in Table 1.

All patients were eligible for a vein bypass. Details of the surgical treatment are presented in Table 2.

## 6. Results

We achieved good results in 789 patients (89.46%). In 93 patients, the results were unsatisfactory. In total, we registered 24 deaths (2.71%), 43 major amputations (4.88%), and 50 cases of clinical decline or lack of clinical improvement (5.67%). As we examined the group with the unsatisfactory outcomes, we found that, for 33 (3.74%) patients, the reason for the unfavorable result was an unfixable technical error; for 48 (5.42%) patients, we found an eligibility error; and 12 (1.36%) cases failed due to other errors. There were 154 errors (17.4%) discovered in total. Of these, 85 were detected on the operating table (including 57 technical errors). Details are presented in Table 3.

Among the 93 errors, 69 were properly detected (true positive) during surgery (7.79%). However, 24 (2.71%) remained undetected (false negative). The efficacy of detecting errors using various peri-operative methods is presented in Table 4.

In discriminant analysis, independent efficacy of error detection was proven only for three of the methods listed above (DSA, DUS, HBF). The test values (Wilks’ lambda; *p*-value) were 0.85, *p* = 0.0000001; 0.62, *p* = 0.011; 0.63, *p* = 0.006, respectively. However, a technical error is not the only factor impacting the early results. Therefore, a discriminant analysis was performed to calculate all factors (listed in Table 1, Table 2 and Table 3), indicating that there are six major factors responsible for compromising early results. These were acute limb ischemia deteriorating to III SVS soon after the surgery (0.83, *p* = 0.0000001), severe cardiovascular complications (0.79, *p* = 0.0000001), major limb amputation in the past (0.77, *p* = 0.0000001), diabetes (0.764, *p* = 0.0001), SVS/ISCVS > 5 (0.763, *p* = 0.0002), and an intraoperatively undetected error (0.756, *p* = 0.017).

**Figure 4:**
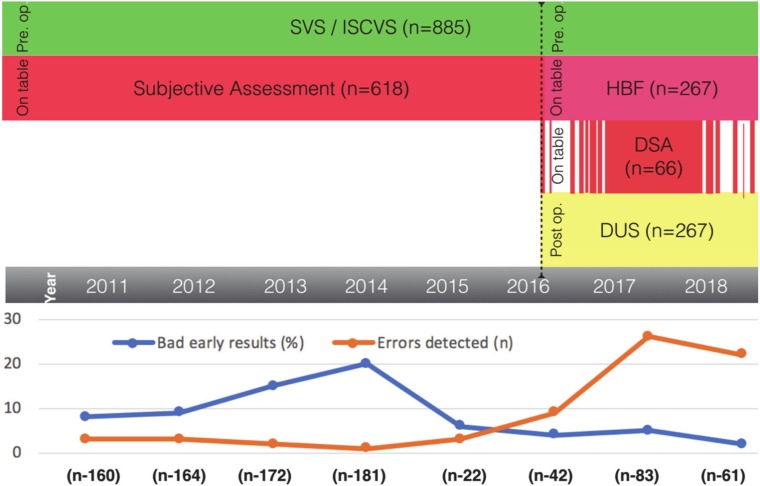
The rising number of detected errors and its influence on the early results.

The introduction of the HBF technique increased the number of intraoperatively detected errors (Figure 4). We noticed a significant improvement of our early results. The percentage of failed cases decreased from 8.6% to 3.7% (*p* = 0.023), and the percentage of detected errors increased from 2.3% to 17.6% (*p* = 0.00001). However, the statistical difference between the control and the examined group decreased in the last few years due to the constantly decreasing number of open bypass surgeries.

The figure shows how the introduction of a new algorithm influenced the results and the rate of error detection. Since the introduction of the HBF technique in 2016, the number of reversible mistakes rapidly increased and the number of failed cases decreased.

## 7. Discussion 

Technical errors are found in about 8–20% of bypass procedures (17.4% in our study). However, they are identified even more frequently if a DUS scan is performed postoperatively [3,5,11]. Like many other authors, we found, that over 50% of errors were related to distal anastomosis failure, and an inadequate outflow was responsible for a further 30% [3]. Technical errors have a major impact on early results. If not corrected intraoperatively, such errors are the most important factor in early graft failure [3]. Therefore, intraoperative monitoring of the bypass function (including the inflow vessel, proximal and distal anastomoses, the conduit, and the distal runoff) is crucial [3].

In our study, intraoperative angiography seemed to be the most specific method for detecting technical errors. Similar findings have led some authors to suggest routine use of angiography after performing a bypass surgery [3,8]. However, such extensive use of angiography is not only expensive, but also time consuming, cumbersome, and carries a certain risk of a contrast-induced nephropathy, especially in cases with a pre-existing kidney dysfunction [14].

Some authors suggested using a detailed intraoperative ultrasound scan [4]. We decided to only use a volume Doppler scan after the operation, to check for bypass thrombosis or compromised flow. Both situations would suggest inflow obstruction or a proximal anastomosis error, which according to our algorithm would require a re-intervention or a more detailed examination (detailed Doppler examination or a CT angiography) before further interventions. In our group, the cutoff for mid-graft PSV was comparatively much lower than that for other references (24 cm/s versus 45 cm/s) [2]. This might be the answer to a problem raised by many authors, as it balances out the potential harm of performing unnecessary procedures on asymptomatic lesions during ultrasound surveillance programs [2].

Subjective methods (based only on the surgeon’s experience) are nowadays no longer applicable. Basing decisions on the surgeon’s “gut feeling” has proved surprisingly effective (possibly more effective than mathematically supported tools) but only for a few surgeons [15].

The SVS/ISCVS score cannot detect technical errors, which may occur during surgery, because it is used preoperatively. It can, however, help with predicting the results. If the SVS/ISCVS score exceeds 6, this should raise the question whether the patient is in fact eligible for the procedure at all (see Table 4). As of today, the SVS/ISCVS score has been replaced by the GLASS score according to the GVG [2].

We introduced the HBF technique to be able to check the bypass flow intraoperatively with (hydrostatic) pressure in a way similar to the Heise technique. Heise et al. [7] used a specially designed system to check the bypass flow. However, the measuring system was complicated and very difficult to repeat. The simplified HBF technique that we propose makes it possible to check the distal anastomoses, the conduit, and the channel the bypass is going through, as well as the distal vessel runoff at the same time. Unfortunately, it is impossible to check the proximal anastomoses with this technique [3]. Hydrostatic pressure in the HBF technique varies between 70 and 100 mmHg depending on the height of the water column. The surgeon must only remember to raise the drip container at least 1 m above the leg to achieve a pressure of 70 mmHg. It is impossible to exceed 100 mmHg because the drip is only 140 cm long. If it is possible to flush 10 mL of saline through the bypass in less than 12 s (meaning that the HBF > 50 mL/min), there are likely no problems with the bypass and a good outflow. If it takes more than 12 s, we suggest looking for a reversible error with the use of intraoperative angiography. In our study, we calculated that the minimal HBF should be at least 53 mL/min, which is similar to the flow reported by Heise (40–60 mL/min in his measuring system) [7]. This made us confident about the accuracy of our results. Moreover, in our suggested technique there is no need for any special devices. Any cannula with the diameters we specified can be used to achieve identical flow parameters. As for the drip set itself, almost any will do, provided it is not longer than 140 cm and has no additional filters, which could provide additional resistance to the cannula.

The HBF technique enables us to detect errors, which are usually difficult to detect by the classic subjective runoff assessment (high-pressure bypass flush performed by the surgeon). We call them discreet occlusions. Checking the flow with high pressure usually does not make it possible to detect rare conditions, such as vein dissection, occlusion of the anastomoses with a valve mechanism, compression, or entrapment of the graft. These errors are difficult to spot using high-pressure techniques but could later become a problem, as the running pressure in the bypass decreases, compromising the actual flow.

Our study proves that the HBF technique is an independent factor that has an impact on the early outcome. Used in an algorithm, together with “on-table” angiography (DSA) and a postoperatively performed ultrasound (DUS) scan, it seems to be the key ingredient in improving results. Different methods of intraoperative runoff checking come with different advantages. We are fully aware of the limitations of our technique (see Table 5).

## 8. Limitations

We are aware of the many limitations of our study. The most important one is the lack of a head-to-head comparison of the methods. This is a result of the chronology of our study and the retrospective methodology. We have been looking for the most effective algorithmic use of various methods together in error detection, rather than assessing their effectiveness separately. This might have triggered the bias of the sensitivity of the DSA. The relatively small number of cases in our study and the fact that DSA was used only as a final check reduced the AUC value in the ROC tests. Angiography remains the most powerful method in the discriminant analysis (as a result of its high specificity—50% TP (true positive) results).

Another bias arises from the study design. Since, the algorithm requires using the SVS/ISCVS score preoperatively and the Doppler scan postoperatively, they are of no use when it comes to scanning for intraoperative errors. The Doppler scan scored high in the ROC tests because it was used to assess the final effect of revascularization, rather than to detect the error. This is why, in the ROC analysis it has the highest sensitivity and specificity. Another bias of the study is associated with the fact that there might be some differences in the height of the water column during the measurement of HBF (ranging from 100 to 140 cm). Those differences result in different pressures being applied in the HBF technique. This is, however, of little significance as the HBF technique is used only for screening for errors causing early obstruction, rather than for predicting the final perfusion. It is crucial that it remains as simple as possible to perform. Therefore, we deliberately decided not to add an additional ruler (or any other water column measuring system) to avoid complicating the technique. Moreover, the maximum length of a drip set is 140 cm. Therefore, the drip set will never be raised above that height and the hydrostatic pressure will never exceed 100 mmHg. The lower the drip set is raised, the lower the pressure, and the longer it will take to flush the saline through the bypass. In the worst-case scenario, this could lead to an increasing number of patients, in which it takes longer than 12 s to flush the saline through the bypass, with the patients therefore requiring additional examinations. This would affect the specificity of the method, but never the sensitivity, which we feel is crucial when it comes to screening methods. Interestingly, in the initial period of the study, we used to check how many times the surgeon would raise the drip set to less than 1 m above the leg, and this never occurred.

False positive results of the HBF technique were observed in cases when the Ancle Brachial Index exceeded 0.7. In those patients, the high pressure in the peripheral vessels compromised the flow during HBF measurement. However, this also affects only the specificity of the method, without influencing its sensitivity (which is more important in screening tests).

Another issue is that the etiologies of disease were very diverse in our patients, however we feel that the analyzed technical errors are not affected by this matter.

All of the listed limitations of our study might be balanced out by a proper use of statistical tools.

## 9. Summary

The HBF technique appears to be a very useful screening tool for “on-table” detection of most technical errors. If the result of HBF is of over 50 mL/min, the patient does not require any additional examinations. The use of the HBF technique, sometimes followed by angiography (according to the algorithm), improves early results. The use of the Doppler scan is important, as it allows to check the quality of the proximal inflow and the anastomoses and must be performed as part of the algorithm after the surgery. The practical relevance of the SVS/ISCVS score is limited. The HBF technique significantly reduces the need for on-table angiography.

## Figures and Tables

**Table 1:** Epidemiology.

Epidemiology		Number/Median	%/SD	Range
Gender				
Male		194	72.66%	
Female		73	27.34%	
Age (years)		65.18	±10.18	50–90
CLTI	Rutherford 4	438	49.49%	
	Rutherford 5	218	24.6%	
	Rutherford 6	229	25.87%	
ABI		0.59	±0.48	0–1.9
Previous vascular interventions		413	46.7%	
Mean number of previous interventions		1.56	±1.07	1–11
V-POSSUM risk of death (%)		2.15%	±2.88	0.2–53
V-POSSUM risk of complications (%)		4.2%	±8.09	0.4–91
Risk of cardiological complication (Goldman-Detsky)		13.77	±8.79	0–50
Hypertension		621	70.17%	
Diabetes		253	28.59%	
Ischemic heart disease		324	36.61%	
Stroke/TIA		130	14.69%	
Contralateral amputation		12	1.36%	
Chronic circulatory failure (NYHA ≥ 2)		111	12.54	
Atrial fibrillation		103	11.64%	
Chronic kidney disease				
I stage		115	19.59%	
II stage		329	56.05%	
III stage		94	16.01%	
IV stage		32	5.45%	
V stage		17	2.9%	
Together: CKD ≥ III		143	24.7%	
Gastric/duodenal ulcer		63	7.12%	
Heavy smokers		306	35.96%	
COPD		70	7.91%	

CLTI—chronic limb-threatening ischemia; ABI—Ankle Brachial Index; V-POSSUM—Vascular-Physiological and Operative Severity Score for the enumeration of Mortality and Morbidity; TIA—Transient Ischemic Attack; NYHA-New York Heart Association; CKD—Chronic Kidney Disease; COPD—Chronic Obstructive Pulmonary Disease.

**Table 2:** Surgical treatment.

Technical Details	Number	%
Vein quality *		
Good	598	67.57
Bad	287	32.42
Types of anastomosis		
Classic (end to side)	838	94.69
End to end	9	10.01
Extra-anatomical (intercostal)	10	1.13
Extra-anatomical (other)	4	0.45
Jump graft on the calf vessels	11	1.24
Y-graft on the calf vessels	13	1.47
Additional procedures		
Endarterectomy of iliac artery	21	2.37
Endarterectomy of common femoral artery	118	13.33
Profundoplasty	123	13.9
Endarterectomy of popliteal artery	28	3.16
Endarterectomy of below the knee	14	1.58
PTA of iliac artery	30	3.39
Necrectomy/small amputation	243	26.44

* Assessed by the surgeon as good if the size of the vein exceeded 4 mm and if no stenosis, varicose veins, or any other condition that might compromise the patency of the graft was present. PTA—Percutaneous Transluminal Angioplasty.

**Table 3:** List of errors and reasons for early failures.

Error Type	Number	%
Complete list of discovered errors	154	17.4
Radiological insufficient “runoff”	29	3.27
Too much tissue loss in critical limb ischemia	20	2.26
Anesthetic complication, hypotension, cardiac arrest, other	12	1.36
Treatment delay and strategy mistake: e.g., lack of fasciotomy	9	1.02
Peripheral embolization	9	1.02
Unrecognized irreversible limb ischemia	6	0.7
Technical errors	69	7.79
Anastomosis error	38	4.29
Iatrogenic damage of target vessels	17	1.92
Vein dissection or torsion	6	1.02
Other mechanical bypass damage	5	0.56
Bypass compression	3	0.34

**Table 4:** Comprehensive error detection efficacy using various methods (ROC analysis).

Examination Method	TP *n* (%)	FN *n* (%)	AUC	Criterion	Sensitivity	Specificity	95% CI	*p*-Value
DUS	37 (29.4%)	3 (2.4%)	0.898	<24 cm/s	80%	80%	0.792–1.0	0.00001
SRA	7 (1.13%)	22 (3.56%)	0.71	<6 pt	65%	60%	0.618–0.802	0.00001
HBF	57 (21.35%)	2 (0.75%)	0.709	<53 mL/min	70%	70%	0.613–0.806	0.00001
SVS/ISCVS	68 (7.68%)	25 (5.8%)	0.685	>6 pt	40%	37%	0.263–0.368	0.00001
DSA	34 (51.51%)	2 (3.03%)	0.494	Visible mistake	NA	NA	0.431–0.556	0.8412

TP—true positive, a detected error; FN—false negative, undetected error; AUC—Area Under Curve; 95% CI—95% confidence interval; DUS—Doppler ultrasound postoperative scan measuring the mid-graft PSV (peak systolic velocity); SRA—subjective runoff assessment (1–8), HBF—hydrostatic bypass flow; SVS/ISCVS—preoperative assessment of SVS/ISCVS scale (1–20) in addition to the Global Limb Anatomic Staging System (GLASS) score; DSA—intraoperative digital subtraction angiography (error/no error visible). The percentage of the TP and FN percentage presented in the table refers to the absolute number of tests presented in Figure 4.

**Table 5:** Comparison of runoff assessment methods.

Advantage	HBF	DSA	SRA	Doppler	SVS/ISCVS
Control of peripheral (1) “runoff”	Yes	Yes	Yes	Yes	Yes
Screen errors intraoperatively	Yes	Yes	Yes (2)	Yes (3)	No
Improve early outcome	Yes	Yes	Yes (2)	Yes (3)	No
Repeatable	Yes	Yes	No	Yes	Yes
Prevent unnecessary reoperation	Yes	Yes	No	No	No
Sensitive to discreet occlusion	Yes	Yes	No	Yes (3)	No
Fast (in performance)	Yes	No	Yes	No	No
Cheap	Yes	No	Yes	No	Yes
Additional equipment not needed	Yes	No	Yes	No	Yes
No contrast needed	Yes	No	Yes	Yes	No
Does not extend operation time	Yes	No	Yes	No	Yes
Easy to learn	Yes	Yes (2)	No	Yes (2)	Yes
Predicting long result	Yes	No	No	Yes	Yes
No risk of infection transfer	Yes (4)	Yes (4)	Yes (4)	No	Yes
Control of inflow and proximal anastomosis	No	Yes	No	Yes	No

Key: (1) means distal anastomosis, graft and its tissue channel, distal runoff vessels; (2) depends on the clinician’s experience; (3) if used intraoperatively; (4) very low; green box—advantage; yellow box—conditional advantage/disadvantage; pink box—disadvantage.
